# Kangfuxinye Enema Combined with Mesalamine for Ulcerative Colitis: A Systematic Review and GRADE Approach

**DOI:** 10.1155/2017/6019530

**Published:** 2017-08-07

**Authors:** Peng-wei Ren, Wen-jie Yang, Dan-dan Wang, Jing-yan Shan, De-ying Kang, Qi Hong, Shu Wen, Ru-wen Zhang

**Affiliations:** ^1^Department of Evidence-Based Medicine and Clinical Epidemiology, West China Hospital, Sichuan University, Chengdu 610041, China; ^2^West China School of Public Health, No. 4 West China Teaching Hospital, Sichuan University, Chengdu 610041, China; ^3^West China School of Medicine and West China Hospital, Sichuan University, Chengdu 610041, China; ^4^Department of Integrated Traditional Chinese and Western Medicine, West China Hospital, Sichuan University, Chengdu 610041, China

## Abstract

**Objectives:**

To critically appraise the efficacy and safety of Kangfuxinye enema combined with mesalamine for the ulcerative colitis (UC) patients and in addition to grade the quality of evidence by using the GRADE (grading of recommendations, assessment, development, and evaluation) approach.

**Methods:**

A literature search was performed in the Cochrane Library, MEDLINE, EMBASE, CBM, CNKI, VIP, and WanFang Databases. The search restrictions were patients with UC and RCTs. Studies including other treatments except Kangfuxinye with mesalamine were excluded.

**Results:**

Nineteen studies met the inclusion criteria. We found significant benefits of Kangfuxinye combined with mesalamine against mesalamine alone in improving response rate as well as reducing the recurrence rate and inflammation rate; meanwhile, the increase of the adverse events rate was not observed. Furthermore, the symptoms remission rate and the cure time were insignificant statistically. Additionally, GRADE results indicated that the quality of evidence regarding the above 6 outcomes was rated from very low to moderate quality.

**Conclusions:**

Although Kangfuxinye enema seems effective and safe for treating UC patients in this systematic review, Kangfuxinye enema combined with mesalamine was weakly recommended due to very low to moderate quality of available evidence by the GRADE approach.

## 1. Introduction

Ulcerative colitis (UC) is one of the 2 major types of inflammatory bowel disease (IBD), along with Crohn disease but 3 times more common compared to it [1, 2]. The incidence of UC is 1.2–20.3 cases per 100,000 per year, and the developed countries, such as Northern Europe and North America, have the highest incidence of the disease [1, 3]. In Asia and the Middle East, the incidence is about 6.3 per 100,000 person-years. Universally, UC occurs mainly between the second and fourth decades of life [4]. In combination with the change of environment and other unknown reasons, UC has become a global emergence disease with increasing incidence and prevalence worldwide [5]. Typical symptoms of UC include abdominal pain, tenesmus, bloody diarrhea, passage of pus, mucus, or both, urgency, weight loss, and fever [6], which causes a miserable influence on the quality of life of the UC patients. Moreover, UC affects individuals in their most formidable and productive years of life, resulting in heavy burden on the patients' life, health care system, and society [7]. In addition, high relapse rates and protracted courses of disease also lead to the increasing risk of colorectal cancer [8, 9]. Therefore, UC often requires life-long maintenance therapy for relieving symptoms and/or to attenuate the inflammation while there is lack of curative treatment.

Mesalamine (USAN), also known as mesalazine (INN, BAN) or 5-aminosalicylic acid (5-ASA), is most commonly used as a first-line therapy for mild to moderate UC [10]. However, the majority of patients with UC exhibited low adherence and persistence to mesalamine, which has been an important barrier for successful management [11]. Indeed, the major consequences of nonadherence to 5-ASA for UC patients had a fivefold higher risk of relapse, an increased risk of colorectal cancer, and a reduced quality of life [8]. Once the first-line therapy fails, patients would turn to alternative medicine such as steroids [12], azathioprine [13], and the antitumour necrosis factor alpha (TNFa) agent infliximab [14]. Nevertheless, those alternative therapies always accompany increased risks of infection and malignancy.

At present, complementary and alternative medicine (CAM) is increasingly applied for treatment of IBD due to its potential efficacy [15, 16], and it accounts for about 21% of inflammatory bowel disease patients now [17]. Of those, Kangfuxinye, a pure Chinese herbal medicine extracted from the* Periplaneta americana*, has been widely used for treating ulcerative and inflammatory diseases [18, 19] due to its sound effects on anti-inflammatory and recovery of gastrointestinal mucosal, and animal studies have also suggested that the therapeutic effect of Kangfuxinye may be due, at least in part, to its stimulatory effect on nonspecific cellular defense mechanisms [20], making it one of the most addressed therapies for UC, especially in Chinese UC patients. Although previous studies had shown sound effects of Kangfuxinye for treating UC patients, the quality of the studies has become a common concern, thus further researches are needed before making recommendations for clinical practice. One previous systematic review (SR) [21] indicated Kangfuxinye having short-term benefits regarding the overall response and inflammation reduction, but its safety and long-term effect still remain unclear. In addition, the quality of evidence needs to be appraised and validated critically.

Therefore, the aims of this study were to systematically review the efficacy and safety of Kangfuxinye enema in combination with mesalamine according to the Cochrane Collaboration's guidance for SR and then to grade quality of the evidence and make recommendations for practice by using The Grading of Recommendations, Assessment, Development and Evaluation (GRADE) approach [22] which is always used as an instrument for grading quality of evidence within systematic reviews and guidelines and for making evidence-based recommendations during guidelines development [23].

## 2. Methods

This study was conducted using the Cochrane Collaboration's approach [43] and this systematic review is consistent with the PRISMA (the Preferred Reporting Items for Systematic Reviews and Meta-Analyses) checklist [44]. In addition, the GRADE approach [22] was also taken to grade the quality of evidence and make recommendation regarding the use of Kangfuxinye enema in the UC. Five methodological factors (risk of bias, inconsistency, indirectness, imprecision, and publication bias) were judged to downgrade or upgrade the quality of evidence [45]. Ethical approval and patient informed consent were waived because all data were extracted from previous studies.

### 2.1. Criteria for Considering Studies for This Review

#### 2.1.1. Type of Studies

Only RCTs, which were published or unpublished in English or Chinese, were identified for this review. Observational studies, quasi-randomized controlled trials (Q-RCTs), controlled clinical trials (CCTs) were excluded.

#### 2.1.2. Types of Participants

Participants (male/female) diagnosed with UC and who met the indications for using Kangfuxinye as enema were included in this study.

#### 2.1.3. Types of Interventions

Kangfuxinye enema combined with mesalamine served as the intervention and taking mesalamine alone served as the control. Any mode of the mesalamine was eligible for this review.

#### 2.1.4. Types of Outcome Measures

We consulted with 5 clinicians specialized in UC from West China hospital, to identify possible outcomes relating to the UC's efficacy and safety as well as to rate clinical importance of each outcome with assigning a value of 1 (lowest importance) to 9 (highest importance). The results were then used to generate a mean score with standard deviation (SD) for each outcome. The importance of each outcome was classified according to the mean score. Three outcome categories were identified regarding the clinical importance: critical (mean score of 7–9), important but not critical (mean score of 4–6), and limited importance (mean score of 1–3) [22]. Critical and important outcomes in Table 1 were used to make recommendations (Table 1).

### 2.2. Search Strategies

#### 2.2.1. Electronic Searches

The following databases were searched from the inception through March 31, 2016: Cochrane Central Register of Controlled Trials (CENTRAL, Ovid), MEDLINE (PubMed), EMBASE (Ovid), Chinese Biomedicine Database (CBM), China National Knowledge Infrastructure (CNKI), VIP Information Database (VIP), and WanFang Database. The search terms used were “Kangfuxinye”; “Mesalamine”; and “ulcerative colitis” in Chinese or English.

#### 2.2.2. Search Other Sources

We also screened reference list of all obtained papers. Additionally, conference proceedings and dissertation abstracts were retrieved to identify unpublished studies.

### 2.3. Selection of Studies

Retrieved records including titles and abstracts were screened independently by 2 reviewers (P-W R and W-J Y) using EndNote 5.0 software after removal of duplications. The studies were included if they were Kangfuxinye enema combined with mesalamine against mesalamine alone. Observational studies, quasi-randomized controlled trials (Q-RCTs), controlled clinical trials (CCTs), and trials with paired interventions besides Kangfuxinye and mesalamine were excluded. Dissertations and abstracts were included when they contained sufficient details. All of the eligible studies were downloaded. Discrepancies were resolved via discussion or in consultation with the third reviewer (D-Y K).

### 2.4. Data Extraction and Management

All studies were reviewed by two reviewers (P-W R, W-J Y), who extracted data from the studies with the predeveloped forms including items such as the following: first author, publication year, sample size in each group, characteristics of participants (including age, sex, and degree of UC), diagnosis criteria of UC, details of Kangfuxinye enema and mesalamine, measured outcomes, follow-up (where available), and the number and reasons of missing participants.

Mean score changes from baseline to a particular endpoint were also abstracted. If unavailable, we extracted mean scores of baseline and endpoint as well as the SDs [43, 46]. Consensus was obtained by discussion or by consulting the third reviewer (D-Y K).

### 2.5. Assessment of Risk of Bias in Included Studies

Risk of bias for each eligible study was assessed by 2 reviewers (P-W R and W-J Y) using the Cochrane Collaboration's Risk of Bias Tool in 6 domains: random sequence generation, incomplete outcome measures, blinding of participants and personnel, and outcome assessors, and allocation concealment, and selective outcome reporting [43]. Disagreements were resolved by discussion between the two reviewers (P-W R, W-J Y), or with the arbitration of a third reviewer (D-Y K) being sought if necessary. There was no disagreement between the two reviewers on the risk of bias.

### 2.6. Data Synthesis and Statistical Analysis

A meta-analysis was performed by using the Review Manager (Version 5.3 for Windows; Cochrane Collaboration, Oxford, UK) if needed. For dichotomous data, pooled effect estimate was calculated using risk ratio (RR) with its 95% confidence interval (CI). For continuous data, overall treatment effect size was calculated using mean difference (MD) with its 95% CI when the same rating scale was used, or using standardized mean difference (SMD) if rating scales were different. A 2-sided *P* ≤ 0.05 was considered as the threshold for statistical significance. Heterogeneity across study results was assessed using Cochrane's *Q* statistic with *P* value. *I*^2^ statistic was used to quantify the degree of heterogeneity. If *P* < 0.1 or *I*^2^ > 50%, this indicates significant heterogeneity was present [43], and a random-effects model was applied to pool overall effect estimate; otherwise, a fixed-effects model was used. Subgroup analyses were carried out where available to investigate potential influence of clinical characteristics of participants or methodological quality on treatment effect size. Sensitivity analyses were performed where available to explore possible heterogeneity and its impact on the robustness of study results. If the number of included studies was sufficient (*n* > 10), a funnel plot or Egger's regression test was generated to detect potential publication bias [47, 48].

### 2.7. The GRADE Approach

Quality of evidence for each specific outcome among the included studies was evaluated by using the GRADE approach. Two authors (P-W R and W-J Y) received training on how to use GRADEro [49] in the 23nd Cochrane Colloquium (Vienna, Austria, from October 3 to 7, 2015), and separately assessed the quality in the estimate of each outcome. The evidence quality across each outcome was upgraded or downgraded determined by 5 primary domains (risk of bias, inconsistency, indirectness, imprecision, and publication bias) and was eventually categorized into 4 levels (high, moderate, low, and very low) [23].

## 3. Results

**Figure 1:**
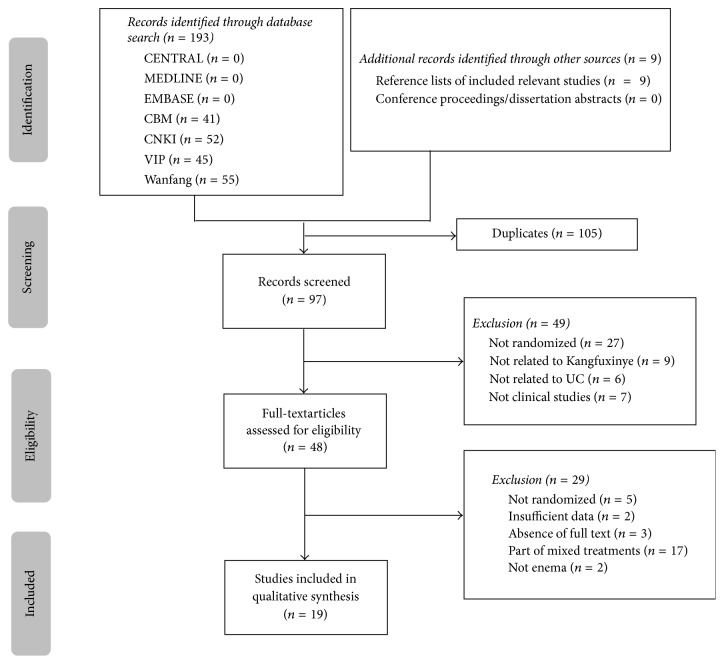
Flow diagram of the selection process. CENTRAL = Cochrane Central Register of Controlled Trials; CBM = Chinese Biomedicine Database; CNKI = China National Knowledge Infrastructure; VIP: Chinese Scientific Journals Database.

Our searches identified 202 potentially relevant studies, of which 193 references were all from electronic databases, 9 references from relevant reference lists, and no references were obtained from conference proceedings or dissertation abstracts. Finally, 19 studies [24–42] from electronic databases met our inclusion criteria. Further details were shown in Figure 1.

### 3.1. Characteristics of Included Studies

The characteristics of all included RCTs [24–42] were listed in Table 2. All RCTs were conducted in China and were published in Chinese. Males approximately account for half of the enrolled patients in each study. No dropouts were observed in these studies.

### 3.2. Assessment of Risk of Bias in Included Studies

The risk of bias of all included RCTs was assessed by using Cochrane Collaboration risk of bias tool. Because of inadequate reporting of randomization sequence generation and allocation concealment, all of the two items were judged as “unclear” which means that the potential risk of selection bias may exist. Of those, only two RCTs [24–42] used random number table to produce random sequence, whereas other trials just reported “randomly assigned” but failed to report on how sequence is produced. Details of allocation being concealed were unclear in all studies. Meanwhile, whether other important risks of bias existed could not be assessed due to paucity of data among the included trials. Overall, the included RCTs had moderate or high risks of bias in terms of 6 domains (Table 3).

### 3.3. Critical Outcomes

#### 3.3.1. Recurrence Rate

**Figure 2:**
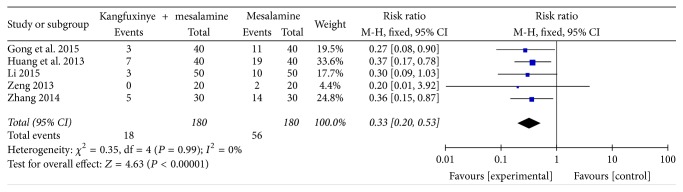
Efficacy of Kangfuxinye combined with mesalamine versus mesalamine on recurrence rate.

Five RCTs [24, 26, 29, 39, 41] including 360 patients reported recurrence rate. Recurrence was monitored after 3~12 months of follow-up among these trials. Compared with mesalamine, the meta-analysis indicated that Kangfuxinye combined with mesalamine enema reduced recurrence significantly (RR = 0.33, 95% CI: 0.20–0.53, *P* < 0.001) without heterogeneity (*I*^2^ = 0%, *P* = 0.99) (Figure 2). A GRADE analysis indicated that the quality of evidence supporting this outcome was moderate due to risk of bias (Table 4).

#### 3.3.2. Response Rate

**Figure 3:**
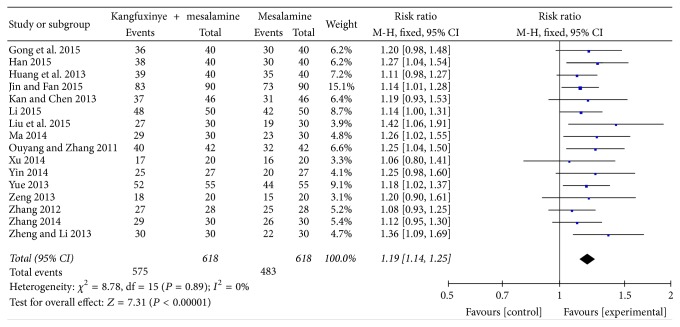
Efficacy of Kangfuxinye combined with mesalamine versus mesalamine on response rate.

16 RCTs [24–31, 33, 36–42] including 1236 patients reported response rate. The outcome measure was based on both physician's assessment and the results of endoscopy typically divided into four categories, including (1) recovery, (2) significant improvement, (3) mild improvement, and (4) no change. The meta-analysis suggested favourable effects of Kangfuxinye combined with mesalamine against mesalamine (RR = 1.19, 95%  CI = 1.14 to 1.25, *P* < 0.0001; heterogeneity: *I*^2^ = 0%, *P* = 0.89) (Figure 3). A GRADE approach indicated that the quality of evidence supporting this outcome was low due to serious risk of bias (Table 4).

### 3.4. Important Outcomes

#### 3.4.1. Inflammation Reduction Rate

**Figure 4:**
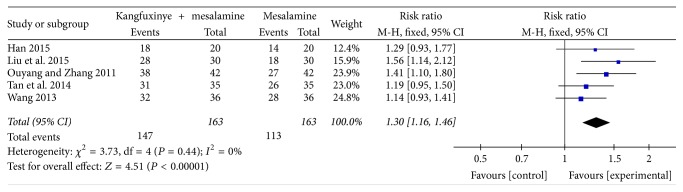
Efficacy of Kangfuxinye combined with mesalamine versus mesalamine on inflammation reduction rate.

Of those included 5 trials [25, 30, 33–35] providing examination of the inflammation reduction by endoscopy and endoscopy grading or scoring systems for inflammatory bowel diseases (IBD), a significant difference on the inflammation reduction rate was observed between two groups (fixed-effects model, RR = 1.30, 95% CI: 1.16–1.46, *P* < 0.001) without heterogeneity (*I*^2^ = 0%, *P* = 0.44) (Figure 4). A GRADE approach indicated that the quality of evidence supporting this outcome was low due to serious risk of bias (Table 4).

#### 3.4.2. Symptom Remission Rate

**Figure 5:**
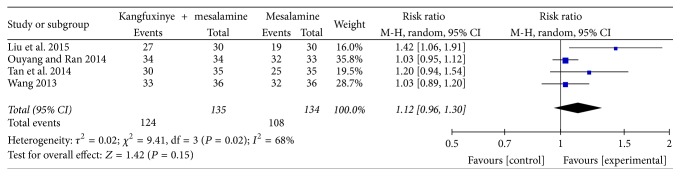
Efficacy of Kangfuxinye combined with mesalamine versus mesalamine on symptom remission rate.

Four studies [30, 32, 34, 35] including 269 patients reported symptom remission rate. The outcome measure was based on both physician's assessment about the patients' general conditions and the patients' feeling. The meta-analysis indicated that no favourable effects of Kangfuxinye combined with mesalamine compared with mesalamine alone were observed (fixed-effect model, RR = 1.12, 95%  CI = 0.96 to 1.30, *P* = 0.15) with moderate heterogeneity (*I*^2^ = 68%, *P* = 0.02) (Figure 5). A GRADE approach indicated that the quality of evidence supporting this outcome was low due to the risk of bias, imprecision, and inconsistency (Table 4).

#### 3.4.3. Time of Remission

**Figure 6:**
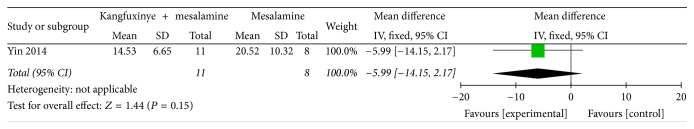
Efficacy of Kangfuxinye combined with mesalamine versus mesalamine on time of remission.

One trial [37] involving 19 participants provided the time of remission, but it failed to present any benefit of Kangfuxinye in terms of shortening time of remission significantly (MD = −5.99, 95% CI: −14.15, 2.17, *P* = 0.15) (Figure 6). A GRADE analysis indicated that the quality of evidence supporting this outcome was very low due to high risk of bias and imprecision (Table 4).

### 3.5. Safety Evaluation

**Figure 7:**
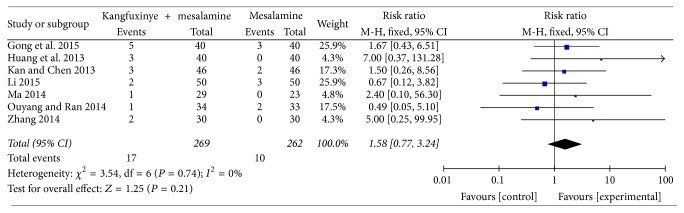
Efficacy of Kangfuxinye combined with mesalamine versus mesalamine on adverse events rates.

Of the 19 RCTs, 7 trials failed to report anything about adverse events, and the other 12 RCTs [24, 26, 28, 29, 31–34, 38, 39, 41, 42] reported adverse events rate. Five trials of those [33, 34, 38, 39, 42] reported no adverse events, while at least 1 adverse event was reported in the other 7 trials [24, 26, 28, 29, 31, 32, 41] which included 451 patients that were taken to explore the safety of Kangfuxinye combined with the mesalamine. The meta-analysis showed no difference in Kangfuxinye combined with mesalamine against mesalamine alone (fixed-effect model, RR = 1.58, 95%  CI = 0.77 to 3.24, *P* = 0.21) without heterogeneity (*I*^2^ = 0%, *P* = 0.74) (Figure 7). A GRADE approach indicated that the quality of evidence supporting this outcome was low due to risk of bias and imprecision (Table 4).

### 3.6. Publication Bias

**Figure 8:**
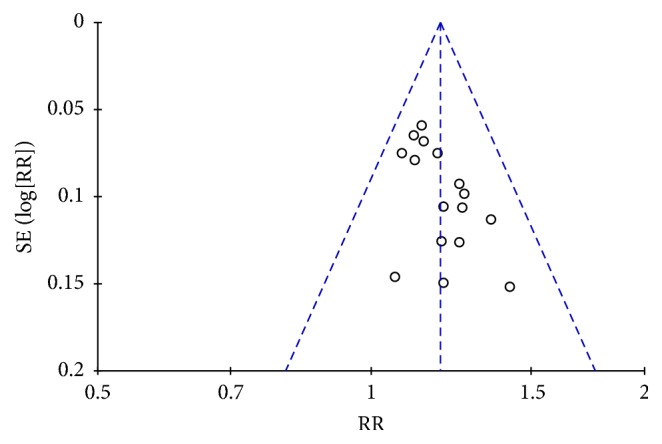
Funnel plot analysis on response rate of the 16 trials comparing Kangfuxinye combined with mesalamine versus mesalamine.

Although an asymmetric funnel plot on the response rate was observed, the Egger et al. [48] test failed to identify any publication bias (*P* = 0.817) (Figure 8).

## 4. Discussion

19 RCTs involving 1685 patients were identified for this study. The results in our study showed that, compared to mesalamine alone, Kangfuxinye enema combined with mesalamine appeared to be more effective either in reducing recurrence rate or in improving response rate and the inflammation reduction rate. With regard to the symptom remission rate, time of remission, and adverse events rate, no significant benefits were observed. A GRADE approach indicated that most of evidences were rated as moderate, low, or very low quality. Compared with the outcome measured in previous systematic reviews, our review rated rank of relative outcomes according to clinical importance. What is more, the recurrence rate ranked as first of those outcomes due to high relapse rate of UC, and the following in descending order was response rate, inflammation reduction rate, symptom remission rate, and time of remission accordingly in the review.

Furthermore, the quality of evidence on the 6 preset outcomes was rated with the GRADE approach. The evidence of each outcome was downgraded one or two levels due to high risk of bias (poor reporting about randomization and allocation concealment), inconsistency, and imprecision. Five of the 6 outcomes were not downgraded in terms of inconsistency and the remission rate of symptoms was downgraded, which may be explained by heterogeneous patients' characteristics, disease cognition, and susceptibility to adverse events. Regarding imprecision, the OIS referred to the number of participants estimated by a sample size calculation for a single adequately powered trial [50]. If the total number of participants of a meta-analysis is lower than the OIS criterion, the quality of evidence should be downgraded because of imprecision [51]. In this study, the 95% CIs of the outcomes of adverse effects rate and symptom remission rate included a relative risk of 1.0 [51]; meanwhile the total number of participants for both outcomes (*n* = 561 and 269, resp.) of the meta-analysis did not exceed conventional sample size (*n* = 1204 and 290) calculation for a single adequately powered trial; therefore it was more likely to support downgrading the evidence quality due to imprecision. In addition, as the sample sizes (*n* = 19) of the time of remission were far less than the OIS (*n* = 300), our confidence in this outcome downgraded two levels. Because there were no significant differences either in baseline characteristics or in the outcomes measured in the included studies, the indirectness was considered as not serious; consequently none of these outcomes was downgraded. Potential publication bias was detected concerning the outcome of inflammation reduction rate through visual inspection. Therefore, the quality of evidence on this outcome was downgraded.

Overall, the quality of evidence with respect to the 6 critical or important outcomes was graded from moderate to very low, and limited data and insufficient follow-up time of long-term effects were more likely to warrant a weak recommendation of Kangfuxinye combined with mesalamine for treating UC patients. Kangfuxinye is crudely extracted by ethanol from dried* P. americana* whole body and has been approved by the China Food and Drug Administration (CFDA) (Z51021834). The main chemical compositions of Kangfuxinye are amino acids, small molecular peptides, and nucleotides. The present study indicated that* P. americana* extract can increase the levels of prostaglandin E2 (PGE2) [52]. And PGE2 can inhibit acid secretion and increase mucosal blood flow, both of which contribute to the repair of gastrointestinal mucosa [53]. Moreover, it also inhibits the release of inflammatory mediators in the gastric mucosa and inhibits neutrophils, monocytes, and macrophages at inflammatory sites [54]. Therefore,* P. americana* has a good effect on the gastrointestinal mucosal repairing and anti-inflammatory. A recent study [55] showed that the abstracts enema could accelerate the healing process in dinitrochlorobenzene (DNCB) and acetic acid- (AA-) induced ulcerative colitis rat, whose symptoms and histological features were similar to those of human UC. Moreover, the mechanism was also confirmed that the abstract of* P. americana* was able to encourage fibroblasts proliferation and collagen synthesis in in vitro fibroblast cell model, NIH 3T3 [55]. And a multitude of clinical researches have reported the positive effect of Kangfuxinye. One previous systematic review [21] concerning the clinical application of Kangfuxinye combined with mesalamine in treating UC patients has found that Kangfuxinye could significantly improve the response rate of the UC. However, with only 11 studies retrieved from the Chinese databases, only 2 outcomes (overall response rate and inflammation reduction rate) were taken to perform the pooled analysis, and the adverse events were not pooled due to unavailable data. Moreover, some of the included studies mixed with other interventions in the combination of mesalamine and Kangfuxinye enema.

In our study, a comprehensive literature search was conducted in 7 electronic databases, and, gray literature databases and references lists were taken to identify relevant studies. We also developed explicit eligibility criteria using PICOS (Participants, Intervention, Comparison, Outcome, Study design) format. Only those that compared Kangfuxinye enema combined with mesalamine against mesalamine alone were included. In addition, we graded 6 critical or important outcomes according to their clinical importance to grade the quality of evidence by GRADE approach and the recurrence rate was taken as the most critical outcome used to explore the long-term effect of Kangfuxinye enema. Furthermore, we explored the safety of Kangfuxinye enema in terms of adverse events rate. By the way, as we searched relevant databases from the inception through March 31, 2016, the conclusion in our review may be recognized more and up to date, comprehensive, and robust. To the best of our knowledge, this is the first systematic review to grade the quality of evidence and then to generate recommendation regarding the use of Kangfuxinye in UC patients. Currently, rating an overall body of evidence by the GRADE approach is becoming an important and recommended explicit step in evidence synthesis initiatives [56]. With this approach, the details of potential limitations, including risk of bias, result inconsistency, indirectness imprecision, and publication bias are scrutinized for every outcome. And the approach provides us with a structured and transparent way to use this evidence for making a recommendation or decision, particularly for the low or unclear quality of evidence [56]. Therefore, it becomes one of the strengths in our study.

Nevertheless, several limitations should be specially addressed before the acceptance of the findings. Firstly, selection bias may occur in the methodological designs of included studies due to the inadequate reporting, although the review processes were appraised rigorously by 2 experienced and independent authors. Secondly, only two trials [24, 26] using a random method divided the groups, and the remaining 17 trials [25, 27–42] reported “randomly allocating” participants but the method of randomization was not described. Thirdly, none of the included trials reported allocation concealment, and whether a blinding method was used or not within 19 trials remains unclear, leading to the increase in risk of selection or performance bias. Last but not least, all included studies were conducted in China and were published in Chinese journals. Although the funnel plot and Egger's regression test failed to detect any publication biases, we could not rule out publication bias absolutely. As studies with statistically significant results are more likely to be published compared to those with null results [57], which seems more common in studies reported in Chinese and other Asian language [58, 59], the pooled RR reported in this study may be exaggerated compared to the true value. It is an important threat to the validity of systematic reviews and is difficult to combat except through the registration of all RCTs. In addition, in most studies, the effect of Kangfuxinye enema would be reduced without full contact with the ulcer on account of the fact that enema position of the patients did not vary according to the ulcer locations in the colon. We also noted that the participants of all the trials were all Chinese and whether it is still effective and could be applied to patients outside of China still needs to be further investigated.

## 5. Conclusions

Kangfuxinye enema addition to mesalamine may be effective and safe for UC patients. As the GRADE approach indicated very low to moderate quality of the evidence and lack of information about patients' preference, we suggest a weak recommendation for Kangfuxinye. Considering that all identified studies were of low quality and all were carried out in China, further rigorously designed and large-scale RCTs outside of China are warranted to improve the generalizability and applicability of this study results. And further GRADE approaches are also needed for grading quality of evidence regarding Kangfuxinye in combination with other additional or alternative medicine for UC patients.

## Supplementary Material

The condition of the manuscript reported according to the PRISMA statement.

## Figures and Tables

**Table 1:** Rating scale for outcome ranking according to clinical importance.

Importance	Measure
Critical^*∗*^	Recurrence rate
	Response rate
Important^†^	Inflammation reduction rate Symptom remission rate
	Adverse effects rate Time of cure
Not important^‡^	None

^*∗*^Critical for making a decision and included in the evidence profile. ^†^Important for making a decision and included in the evidence profile. ^‡^Not important for making a decision and not included in the evidence profile.

**(a):** 

Studies	Number of participants		Age (mean ± SD/range, y)		Sex (male, %)		Degree of UC	Disease course (mean ± SD/range, y)		Diagnosis
	Experiment	Control	Experiment	Control	Experiment	Control		Experiment	Control	
Gong 2015 [24]	40	40	58.1 ± 8.2	58.4 ± 9.0	45.0	45.0	Mild to moderate	3.6 ± 1.6	3.3 ± 1.4	WGOPGDMIBD (revised 2010)
Han 2015 [25]	20	20	44 ± 13.1	43.5 ± 12.3	35.0	35.0	NR	4.8 ± 2.5	4.5 ± 2.3	NR
Huang 2013 [26]	40	40	67.1 ± 10.4	68.5 ± 8.6	42.5	50.0	Mild to severe	NR	NR	WGOPGDMIBD (revised 2010)
Jin 2015 [27]	90	90	45.2 ± 3.7	45.3 ± 3.5	54.4	54.4	NR	3.8 ± 1.4	3.5 ± 1.3	CTCMDTUC (revised 2010)
Kan 2013 [28]	46	46	20~74		NR	NR	NR	NR	NR	CCDTIBD (revised 2007)
Li 2015 [29]	50	50	34.9 ± 6.2	34.5 ± 6.5	66.0	66.0	NR	2.4 ± 0.7	2.5 ± 1.0	WGOPGDMIBD (revised 2010)
Liu 2015 [30]	30	30	24~62	27~63	40.0	40.0	NR	1~8	0.75~6	CCDTIBD (revised 2007)
Ma 2014 [31]	30	30	20~50	17~50	60.0	60.0	Mild to moderate	0.5~2	0.25~2	NR
Ouyang 2014 [32]	34	33	36.3 ± 5		NR	NR	Mild to moderate	NR	NR	CCDTIBD (revised 2007)
Ouyang 2011 [33]	42	42	20~78		NR	NR	Mild to moderate	NR	NR	NR
Tan 2014 [34]	35	35	43.5 ± 10.4	45.2 ± 11.3	54.3	54.3	NR	0.5~16	0.5~14	WGOPGDMIBD (revised 2010)
Wang 2013 [35]	36	36	18~70		NR	NR	Mild to moderate	NR	NR	CCDTIBD (revised 2007)
Xu 2014 [36]	20	20	23~55		NR	NR	Mild to moderate	NR	NR	NR
Yin 2014 [37]	27	27	51.02 ± 3.14	50.20 ± 3.03	48.1	48.1	Moderate to sever	5.49 ± 1.45	5.93 ± 1.57	NR
Yue 2013 [38]	55	55	20~74	19~74	41.8	41.8	Mild to moderate	8.5 ± 0.9	8.2 ± 0.8	CCDTIBD (revised 2012)
Zeng 2013 [39]	20	20	46.0 ± 3.5	45.0 ± 2.5	40.0	40.0	Mild to moderate	9.5 ± 0.5	8.5 ± 1.5	CCDTIBD (revised 2007)
Zhang 2012 [40]	28	28	NR	NR	NR	NR	NR	NR	NR	NR
Zhang 2014 [41]	30	30	45 ± 12	47 ± 11	46.7	46.7	Mild to moderate	NR	NR	NR
Zheng 2013 [42]	30	30	22~63	29~58	63.3	63.3	NR	0.7~14	0.9~9	CCDTIBD (revised 2007)

**(b):** 

Studies	Intervention strategy	Control strategy	Course	Adverse events		Recurrence		Follow-up time (month)
				Experiment	Control	Experiment	Control	
Gong 2015 [24]	KFXY enema (50 ml of KFX in 150 ml of normal saline; 37°C) + mesalamine Enteric-coated Tablets 1.0 g tid	Mesalamine Enteric-coated Tablets 1.0 g tid	NR	5	3	3	11	6
Han 2015 [25]	KFXY enema (50 ml of KFX in 150 mL of normal saline; 37°C) + mesalamine 1.0 g qid	Mesalamine 1.0 g qid	NR	NR	NR	NR	NR	NR
Huang 2013 [26]	KFXY enema (30 ml of KFX in 150 ml of normal saline; 37°C; 20 min) + mesalamine 1.0 g qid	Mesalamine 1.0 g qid	4 w	3	0	7	19	12
Jin 2015 [27]	KFXY enema (50 ml of KFX in 150 mL of normal saline; 37°C) + mesalamine 1.5–4.0 g/day	Mesalamine 1.5–4.0 g/day	4 w	NR	NR	NR	NR	NR
Kan 2013 [28]	KFXY enema (50 ml of KFX in 50 mL of normal saline; 37°C) + mesalamine Slow Release Tablets 1.0 g qd	Mesalamine Slow Release Tablets 1.0 g qd	4 w	3	2	NR	NR	NR
Li 2015 [29]	KFXY enema (50 ml of KFX in 50 mL of normal saline; 37°C; 45 min) + mesalamine Slow Release Tablets 1.0 g qd	Mesalamine Slow Release Tablets 1.0 g qd	4 w	2	3	3	10	3
Liu 2015 [30]	KFXY enema (100 ml; 20 min) + mesalamine 1.0 g tid	Mesalamine 1.0 g tid	8 w	NR	NR	NR	NR	NR
Ma 2014 [31]	KFXY enema (38–41°C; 45 min) + mesalamine Slow Release Tablets 1.0 g tid	Mesalamine Slow Release Tablets 1.0 g tid	4 w	1	0	NR	NR	NR
Ouyang 2014 [32]	KFXY enema (30 ml of KFX in 120 mL of normal saline; 37°C; 45 min) + mesalamine Slow Release Tablets 1.0 g qd	Mesalamine Slow Release Tablets 1.0 g tid	4 w	1	2	NR	NR	NR
Ouyang 2011 [33]	KFXY enema (50 ml of KFX in 50 mL of normal saline) + mesalamine 1.0 g tid	Mesalamine 1.0 g tid	4 w	0	0	NR	NR	NR
Tan 2014 [34]	KFXY enema (50 ml of KFX in 150 mL of normal saline; 37-38°C) + mesalamine 1.0 g tid	Mesalamine 1.0 g tid	4 w	0	0	NR	NR	NR
Wang 2013 [35]	KFXY enema (50 ml of KFX in 100 mL of normal saline) + mesalamine 1.0 g qid	Mesalamine 1.0 g tid	4 w	NR	NR	NR	NR	NR
Xu 2014 [36]	KFXY enema (50 ml of KFX in 150 mL of normal saline; 37°C) + mesalamine 1.0 g qid	Mesalamine 1.0 g tid	NR	NR	NR	NR	NR	NR
Yin 2014 [37]	KFXY enema (50 ml of KFX in 50 mL of normal saline) + mesalamine 1.0 g tid	Mesalamine 1.0 g tid	4 w	NR	NR	NR	NR	NR
Yue 2013 [38]	KFXY enema (50 ml of KFX in 50 mL of normal saline; 38°C; 45 min) + mesalamine 1.0 g tid	Mesalamine 1.0 g tid	4 w	0	0	NR	NR	NR
Zeng 2013 [39]	KFXY enema + mesalamine 1.0 g tid	Mesalamine 1.0 g tid	NR	0	0	0	2	6
Zhang 2012 [40]	KFXY enema + mesalamine 1.0 g tid	Mesalamine 1.0 g tid	2 w	NR	NR	NR	NR	NR
Zhang 2014 [41]	KFXY enema (50ml of KFX in 150 ml of normal saline; 37-38°C) + mesalamine 1.0 g tid	Mesalamine 1.0 g tid	4 w	2	0	5	14	12
Zheng 2013 [42]	KFXY enema (50 ml of KFX in 100 ml of normal saline; 37.5°C; >30 min) + mesalamine 1.0 g tid	Mesalamine 1.0 g tid	2 w	0	0	NR	NR	NR

WGOPGDMIBD = World Gastroenterology Organization Practice Guidelines for the Diagnosis and Management of IBD, CTCMDTUC = Consensus of Traditional Chinese Medicine Diagnosis and Treatment for Ulcerative Colitis. CCDTIBD = Chinese Consensus on the Diagnosis and Treatment of Inflammatory Bowel Disease (IBD). NR = not reported. KFXY = Kangfuxinye.

**Table 3:** Assessment of risk of bias in included studies.

Studies	Random sequence generation	Allocation concealment	Blinding of participants and personnel	Blinding of outcome assessment	Incomplete outcome data	Selective reporting	Other sources of bias
Gong 2015 [24]	Random number table	Unclear	Unclear	Unclear	Yes	Yes	Unclear
Han 2015 [25]	Unclear	Unclear	Unclear	Unclear	Yes	Yes	Unclear
Huang 2013 [26]	Random number table	Unclear	Unclear	Unclear	Yes	Yes	Unclear
Jin 2015 [27]	Unclear	Unclear	Unclear	Unclear	Yes	Yes	Unclear
Kan 2013 [28]	Unclear	Unclear	Unclear	Unclear	Yes	Yes	Unclear
Li 2015 [29]	Unclear	Unclear	Unclear	Unclear	Yes	Yes	Unclear
Liu 2015 [30]	Unclear	Unclear	Unclear	Unclear	Yes	Yes	Unclear
Ma 2014 [31]	Unclear	Unclear	Unclear	Unclear	Yes	Yes	Unclear
Ouyang 2014 [32]	Unclear	Unclear	Unclear	Unclear	Yes	Yes	Unclear
Ouyang 2011 [33]	Unclear	Unclear	Unclear	Unclear	Yes	Yes	Unclear
Tan 2014 [34]	Unclear	Unclear	Unclear	Unclear	Yes	Yes	Unclear
Wang 2013 [35]	Unclear	Unclear	Unclear	Unclear	Yes	Yes	Unclear
Xu 2014 [36]	Unclear	Unclear	Unclear	Unclear	Yes	Yes	Unclear
Yin 2014 [37]	Unclear	Unclear	Unclear	Unclear	Yes	Yes	Unclear
Yue 2013 [38]	Unclear	Unclear	Unclear	Unclear	Yes	Yes	Unclear
Zeng 2013 [39]	Unclear	Unclear	Unclear	Unclear	Yes	Yes	Unclear
Zhang 2012 [40]	Unclear	Unclear	Unclear	Unclear	Yes	Yes	Unclear
Zhang 2014 [41]	Unclear	Unclear	Unclear	Unclear	Yes	Yes	Unclear
Zheng 2013 [42]	Unclear	Unclear	Unclear	Unclear	Yes	Yes	Unclear

Yes = low risk of bias; No = high risk of bias; unclear = uncertain risk of bias.

**Table 4:** Assessment of quality and summarizing the findings with the GRADE approach.

Quality assessment							Summary of findings				
Participants (studies) Follow-up	Risk of bias	Inconsistency	Indirectness	Imprecision	Publication bias	Overall quality of evidence	Study event rates (%)		Relative effect (95% CI)	Anticipated absolute effects	
							With control	With Kangfuxinye + mesalazine		Risk with control	Risk difference with Kangfuxinye + mesalazine (95% CI)
*Recurrence rate* (critical outcome)											
360 (5 studies) 8 months	Serious^1^	No serious inconsistency	No serious indirectness	No serious imprecision^2^	Undetected^3^	⊕⊕⊕⊝ *MODERATE*^1,2,3^ due to risk of bias	56/180 (31.1%)	18/180 (10%)	*RR 0.33* (0.2 to 0.53)	*Study population*	
										*311 per 1000*	*208 fewer per 1000* (from 146 fewer to 249 fewer)
										*Moderate*	
										*275 per 1000*	*184 fewer per 1000* (from 129 fewer to 220 fewer)

*Response rate* (critical outcome)											
1236 (16 studies) 4 weeks	Very serious^4^	No serious inconsistency	No serious indirectness	No serious imprecision^5^	Undetected	⊕⊕⊝⊝ *LOW*^4,5^ due to risk of bias	483/618 (78.2%)	575/618 (93%)	*RR 1.19* (1.14 to 1.25)	*Study population*	
										*782 per 1000*	*148 more per 1000* (from 109 more to 195 more)
										*Moderate*	
										*764 per 1000*	*145 more per 1000* (from 107 more to 191 more)

*Inflammation reduction rate* (important outcome)											
326 (5 studies) 4 weeks	Very serious^6^	No serious inconsistency	No serious indirectness	No serious imprecision^7^	Undetected^3^	⊕⊕⊝⊝ *LOW*^3,6,7^ due to risk of bias	113/163 (69.3%)	147/163 (90.2%)	*RR 1.3* (1.16 to 1.46)	*Study population*	
										*693 per 1000*	*208 more per 1000* (from 111 more to 319 more)
										*Moderate*	
										*700 per 1000*	*210 more per 1000* (from 112 more to 322 more)

*Symptom remission rate* (important outcome)											
269 (4 studies) 4 weeks	Very serious^8^	Serious^9^	No serious indirectness	Serious^10^	Undetected^3^	⊕⊝⊝⊝ *VERY LOW*^3,8,9,10^ due to risk of bias, inconsistency, imprecision	108/134 (80.6%)	124/135 (91.9%)	*RR 1.12* (0.96 to 1.3)	*Study population*	
										*806 per 1000*	*97 more per 1000* (from 32 fewer to 242 more)
										*Moderate*	
										*802 per 1000*	*96 more per 1000* (from 32 fewer to 241 more)

*Time of remission* (important outcome; better indicated by lower values)											
19 (1 study) 4 weeks	Serious^11^	No serious inconsistency	No serious indirectness	Very serious^12^	Undetected^3^	⊕⊝⊝⊝ *VERY LOW*^3,11,12^ due to risk of bias, imprecision	8	11	*MD-5.99* (−14.15 to 2.17)		The mean time of cure in the intervention groups was *5.99 lower* (14.15 lower to 2.17 higher)

*Adverse effects rate* (important outcome)											
531 (7 studies) 4 weeks	Serious^13^	No serious inconsistency	No serious indirectness	Serious^14^	Undetected^3^	⊕⊕⊝⊝ *LOW*^3,13,14^ due to risk of bias, imprecision	10/262 (3.8%)	17/269 (6.3%)	*RR 1.58* (0.77 to 3.24)	*Study population*	
										*38 per 1000*	*22 more per 1000* (from 9 fewer to 85 more)
										*Moderate*	
										*44 per 1000*	*26 more per 1000* (from 10 fewer to 99 more)

^1^Only 2 studies used random number table to generate random sequence, whereas the 3 remaining trials just reported “randomly assigned” but no mention was made of sequence. Details on how allocation was concealed were unclear in these studies. ^2^The 95% CI excluded a relative risk of 1.0 and the sample size (*n* = 360) met the optimal information size (OIS) criterion, which was calculated approximately as 114. ^3^It was impossible to check publication bias because of limited number of trials for this outcome. ^4^Only 2 studies used random number table to generate random sequence, whereas the 14 remaining trials just reported “randomly assigned” but no mention was made of sequence. Details on how allocation was concealed were unclear in these studies. ^5^The 95% CI excluded a relative risk of 1.0 and the sample size (*n* = 1236) met the optimal information size (OIS) criterion, which was calculated as 176. ^6^All of the 5 trials just reported “randomly assigned” but no mention was made of sequence. Details on how allocation was concealed were unclear in these studies. ^7^The 95% CI excluded a relative risk of 1.0 and the sample size (*n* = 326) met the optimal information size (OIS) criterion,  which was calculated approximately as 114. ^8^All of the 4 trials just reported “randomly assigned” but no mention was made of sequence. Details on how allocation was concealed were unclear in these studies. ^9^Inconsistencies were found among the 4 studies in the pooled results with a considerable heterogeneity (*I*^2^ = 68%, *P* < 0.05). ^10^The 95% CI included a relative risk of 1.0 and the sample size (*n* = 269) failed to meet the optimal information size (OIS) criterion, which was calculated approximately as 290. ^11^This study just reported “randomly assigned” but no mention was made of sequence. Details on how allocation was concealed were unclear in these studies. ^12^Sample sizes and number of events (*n* = 19) were far less than the number of patients generated by a conventional sample size (*n* = 300) calculation for a single adequately powered trial, and the change of our confidence for this outcome was very serious, thus downgrading.^13^Only 2 studies used random number table to generate random sequence, whereas the 5 remaining trials just reported “randomly assigned” but no mention was made of sequence. Details on how allocation was concealed were unclear in these studies. ^14^The 95% CI included a relative risk of 1.0 and the sample size (*n* = 531) failed to meet the optimal information size (OIS) criterion, which was calculated approximately as 1204.
